# Prefrontal activation in suicide attempters during decision making with emotional feedback

**DOI:** 10.1038/s41398-020-00995-z

**Published:** 2020-09-18

**Authors:** Adrián Alacreu-Crespo, Emilie Olié, Emmanuelle Le Bars, Fabienne Cyprien, Jérémy Deverdun, Philippe Courtet

**Affiliations:** 1 Department of Emergency Psychiatry and Acute Care, CHU Montpellier, Montpellier, France; 2 PSNREC, Univ Montpellier, INSERM, CHU de Montpellier, Montpellier, France; 3 Department of Neuroradiology, Academic hospital of Montpellier & U1051, Institut of Neurosciences of Montpellier, Montpellier, France; 4 I2FH, Institut d’Imagerie Fonctionnelle Humaine, Montpellier University Hospital, Gui de Chauliac Hospital, Montpellier, France

**Keywords:** Predictive markers, Psychiatric disorders, Neuroscience

## Abstract

Emotional feedback, such as faces showing emotions, can influence decision making. Decision making and emotional face processing, mainly mediated by the prefrontal and cingulate cortices, are impaired in suicide attempters. Here, we used functional MRI (fMRI) to study prefrontal activation in suicide attempters during a modified version of the Iowa Gambling Task (IGT) that included emotional face feedback. We randomly distributed the 116 euthymic women (*n* = 45 suicide attempters, *n* = 41 affective controls with history of depression without suicide attempt, and *n* = 30 healthy controls) included in the study in three emotional IGT groups: concordant (safe and risky choices followed by happy and angry faces, respectively), discordant (safe and risky choices followed by angry and happy faces, respectively), and neutral condition (safe and risky choices followed by neutral faces). Considering the two IGT phases (ambiguous and risky), we then analyzed five regions of interest during the risky vs. safe choices: orbitofrontal (OFC), anterior cingulate (ACC), ventrolateral (VLPFC), medial (MPFC) and dorsal prefrontal (DPFC) cortices. We found: (1) impaired decision making and increased DPFC and OFC activation in suicide attempters vs. controls in the discordant condition during the risky phase; (2) reduced VLPFC activation in suicide attempters in the concordant condition during the ambiguous phase; and (3) decreased OFC, ACC and DPFC activation in both control groups in the concordant condition during the ambiguous phase. Suicide attempters showed prefrontal alterations during reward-learning decision making with emotional feedback. Suicide attempters may guide their decisions to avoid social negative feedback despite the expected outcome.

## Introduction

Suicidal acts may be viewed as the outcome of dysfunctional decision making. In a phenomenological qualitative analysis that included eight suicide attempters, Bergman et al.^1^ reported a precarious decision-making state about their own life and destiny. Several studies that used the Iowa Gambling Task (IGT)^2^ found that decision making is impaired in subjects with history of suicidal acts, but not suicidal ideation^3–10^. IGT is a decision-making task with monetary feedback after each choice. This feedback should allow participants to learn which choices are safe (i.e., larger wins than losses) and risky (i.e., larger losses than wins) for earning money during 100 trials^11^. IGT is a decision-making task that involves value-based learning and presents two degrees of uncertainty: an ambiguous phase when subjects cannot estimate the outcome (first 40 trials), and a risky phase when subjects can better estimate the possible outcome (last 60 trials)^12,13^. Compared with patients without history of suicide attempt, decision-making is impaired in the risky phase of the IGT in suicide attempters^4,7–10^.

Some neuroimaging studies have been performed to identify the neural bases of decision making as a suicidal vulnerability trait. Jollant et al.^14^ performed functional magnetic resonance imaging (fMRI) during the IGT and found that orbitofrontal cortex (OFC) activation was decreased during risky choices (relative to safe choices) in euthymic suicide attempters compared with patients without suicide history. In a replication study, Olié et al.^15^ showed that dorsal prefrontal cortex (DPFC) activation was decreased during the risky compared with safe choices in suicide attempters. They also reported increased OFC, DPFC and anterior cingulate cortex (ACC) activation during monetary wins relative to losses in suicide attempters compared with non-attempters. Furthermore, differences in OFC and DPFC activation have been observed in relatives and non-relatives of suicide completers during the IGT risky choices compared with safe choices^16^.

During value-based decision making, OFC role is to assess the risk level depending on the reward value^17^. Conversely, DPFC is implicated in the cognitive control of actions and in the integration of complex rules^18,19^. Therefore, it has been hypothesized that both emotional and motivational processes are involved in the decision-making impairment observed in suicide attempters^20^.

Every day people are confronted with a variety of emotional/social cues and they must interpret their underlying meaning in function of the context. According to the somatic marker hypothesis^21^, emotional feedback and the related physiological activations act as signals that might guide future choices. Some studies demonstrated that emotional feedback can bias decision-making processes, even in the presence of monetary feedback^22–24^. More concretely, in the IGT, healthy people with congruent monetary/emotional feedback (happy faces) chose more safe decks than risky decks compared to subjects with incongruent feedbacks (fearful faces) or no feedback^25^. In addition, suicide attempters have difficulties in interpreting and integrating emotional/social signals. For instance, when viewing angry faces relative to neutral faces, activation of OFC and ventrolateral prefrontal cortex (VLPFC) is increased and DPFC activation is decreased in suicide attempters compared with patients without history of suicidal acts.^15,26^. Moreover, ACC activation is increased in suicide attempters when viewing happy faces relative to neutral faces^15,26^. This suggests that suicide attempters might be more sensitive to negative than positive emotional cues. This hypothesis leads to the question of whether suicide attempters would benefit differently from emotional feedback during decision-making processes compared with non-attempters.

To address this question, we used fMRI to compare brain activation in patients with and without lifetime history of suicide attempts and in healthy controls (reference group) during a modified version of the IGT. To avoid gender bias and the effect of acute depression, we recruited only euthymic women. This modified version of the IGT included emotional feedback using angry, happy and neutral faces that were shown between the card choice and the monetary feedback. The emotional feedback given by these images might modulate the decision-making performances. Based on previous research, we hypothesized that suicide attempters would not have the same behavioral benefits from emotional feedback than affective and healthy controls, and that this would be mirrored also by different OFC and DPFC activation profiles.

## Methods and materials

### Participants

This study enrolled 116 euthymic women, aged between 19 and 54 years (mean ± SEM = 36.96 ± 0.82). This sample was used in a previous fMRI study on brain processing of social rejection^27^. Participants were included if they met the inclusion criteria indicated below, and then were screened in person by a psychiatrist. All women were Caucasian, right-handed (Edinburgh Handedness Inventory)^28^, and euthymic at the time of the fMRI scan, as indicated by their Hamilton Depression Rating Scale (HDRS)^29^ and Young Mania Rating Scale (YMRS)^30^ scores (both < 7). Exclusion criteria were: lifetime history of severe head trauma, central nervous system disorder, schizophrenia, history of alcohol or drug abuse or dependence within the past 12 months, pregnancy, and contraindications to MRI.

Participants were divided in three groups: patients with past history of major depressive episode and suicide attempt (suicide attempters = SA; *n* = 45), patients with history of depressive episode without suicidal behavior (affective controls = AC; n = 41), and subjects without psychiatric history (healthy controls = HC; *n* = 30). A suicide attempt was defined as a self-damaging act carried out with certain intention to die. It is different from self-mutilation, the use of substances, or non-compliance with medical treatments^31^. Diagnoses were made according to the DSM-IV criteria using the Mini-International Neuropsychiatric Interview 5.0 (MINI)^32^.

Patients were recruited among the outpatients of the Department of Emergency Psychiatry & Post-Acute Care of Montpellier Academic Hospital (France). HCs were recruited through advertisement and from a list of volunteers in the Montpellier Academic Hospital database.

The Montpellier University Hospital Ethics Committee (CPP Sud Mediterranée IV) approved the study. A signed informed written consent was obtained from all participants. All participants received 100€ for their participation in the study.

### Clinical assessment

In the week preceding the fMRI, the following data were collected; (1) sociodemographic characteristics and medication intake using ad-hoc structured interviews, (2) depressive symptomatology using the Beck Depression Inventory (BDI)^33^, (3) state and trait anxiety using the State-Trait Anxiety Inventory (STAI)^34^, (4) impulsivity using the Barrat Impulsiveness Scale (BIS-10)^35^, (5) verbal intelligence quotient (IQ) using the French version of the National Adult Reading Task (NART)^36^, (6) and past history of childhood trauma using the Childhood Trauma Questionnaire (CTQ)^37^.

### Modified IGT

The IGT models real-life decision making because participants are initially unaware of the underlying contingencies. Specifically, two decks are long-term advantageous (“safe decks”) because wins are low but losses are lower, leading to a net gain. The other two decks are disadvantageous (“risky decks”) because wins are high but losses higher, leading to a net loss. Each choice leads to a gain or a loss of a variable money amount. The goal of the game is to win as much money as possible. Individuals learn from experience to avoid the risky decks. An IGT modified for fMRI studies^2,38^ was used. Four decks of cards (two safe and two risky) were presented on a screen. Participants were prompted to pick a card from the deck of their choice (“Pick a card”). After each choice, a positive or negative amount of virtual money (win or loss) was shown for 2 s. The IG index was the number of choices from the safe decks minus those from the risky decks for all 100 choices. The number of choices from the safe decks minus those from risky decks for trials 1–40 and from trials 41–100 represented the IG ambiguous (i.e., decision making under ambiguous uncertainty) and IG risk (i.e., decision making under risk uncertainty), respectively. After the task completion, the explicit understanding of the task contingencies was rated from 0 (none) to 2 (completely understood).

**Fig. 1:**
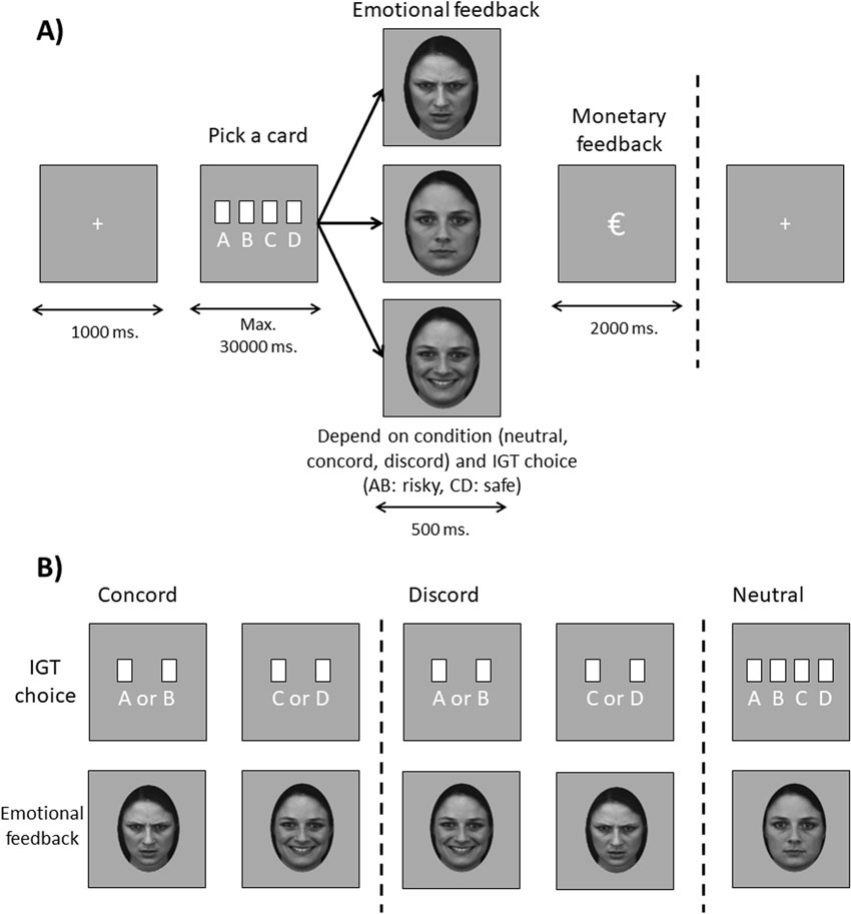
Modified version of the Iowa Gambling Task (IGT). **a** fMRI during the modified IGT with different (neutral, concordant, discordant) emotional feedback relative to the choice. **b** Summary of the different emotional feedback conditions (**a**, **b**: risky decks; **c**, **d**: safe decks of cards). Note: Concord concordant, Discord discordant.

In our modified version of the IGT, emotional feedback (i.e., pictures of angry, happy or neutral faces previously used by the team^15^) was added between the choice (card picking) and the monetary feedback to create three different feedback conditions: concordant (safe and risky choices followed by happy and angry faces, respectively), discordant (safe and risky choices followed by angry and happy faces, respectively), and neutral condition (safe and risky choices followed by neutral faces) independently of the monetary gains or losses. Figure 1 summarizes the procedure.

Within the three groups, participants were randomly distributed as follows: SA group (*n* = 14 in the neutral, *n* = 16 in the concordant, and *n* = 15 in the discordant condition), AC group (*n* = 13 in the neutral, *n* = 15 in the concordant, and *n* = 13 in the discordant condition), and HC group (*n* = 10 in the neutral, *n* = 11 in the concordant, and *n* = 9 in the discordant condition). All participants were task-naïve.

### Image acquisition

fMRI images were acquired at the Neuroradiology Department - I2FH (Academic Hospital of Montpellier) - using a 1.5 T whole-body MRI system (MAGNETON AVANTO, Siemens, Erlangen, Germany) equipped with a standard 12-channel receive-only head coil. Image acquisition during the IGT task was performed using gradient echo-echo planar imaging (GE-EPI) with the following parameters: TR = 3 s, TE = 40 ms, FOV = 220 mm, 42 slices, voxel size = 3.75 × 3.75 × 3.3 mm, flip angle 90°, 403 volumes.

An additional 3D magnetization-prepared, rapid acquisition gradient echo (MP-RAGE) sequence was also obtained for each participant with the following parameters: TR = 2100 ms, TE = 4.1 ms, IR = 1100 ms, 15° flip angle, PAT = 2, aligned with the corpus callosum, voxel-size 0.98 × 0.98 × 1 mm, 160 transversal slices.

### Statistical analysis

#### fMRI data analysis

Data were analyzed using SPM12 (Wellcome Department of Imaging Neuroscience, London, UK) and Matlab R2019a (Mathworks, Inc., Natick, MA). The first four volumes of each fMRI run were discarded due to the time needed to launch the IGT synchronized with fMRI acquisition. Then, the first 40 trials corresponded to the Ambiguous and the others to the Risky phase of the IGT. GE-EPI data were re-oriented to the anterior commissure, slice-time corrected, realigned to the first volume, co-registered, spatially normalized (based on T1) using the DARTEL algorithm, and smoothed with an 8-mm FWHM Gaussian filter.

Contrast images were computed for risky vs. safe choices for each participant using a first-level general linear model. Realignment parameters were added in the regressor to remove specific activation of head movements and a high-pass filter (cut-off = 128 s) was used to remove non-physiological slow signal shifts. A second-level analysis was then performed using a factorial design to evaluate the interaction between Group (SA, AC, HC) and Condition (Neutral, Concordant, Discordant) separately for the two IGT phases (Ambiguous and Risky). For the voxel wise analysis, significance threshold was set at *p* < 0.001 (uncorrected), with *k* ≥ 10 voxels.

Specific regions of interest (ROI) were chosen on the basis of an a priori hypothesis concerning the specific anatomical brain regions that could be associated with decision making, as done in Olié et al.^15^. The ROIs were anatomically defined by Anatomical Automatic Labeling (AAL), using masks provided by the Wake Forest University PickAtlas software (http://fmri.wfubmc.edu). OFC was defined as the orbital parts of the inferior frontal gyrus, middle frontal gyrus and medial frontal gyrus (including the medial and lateral parts) (corresponding to the Brodmann areas (BA) 11/47), VLPFC as the opercular part of the inferior frontal gyrus (BA 44/45), MPFC as the medial part of the superior frontal gyrus (BA 10), and ACC (BA 24/32), and DPFC as the middle frontal gyrus (BA 8/9/46) (Supplementary Fig. 1).

#### Clinical and behavioral data analysis

The variable normality was checked with the *Kolmogorov–Smirnov* test and homogeneity of variances with the *Levene* test; non-normal variables were log transformed. Preliminary ANOVAs or Student’s *t* tests (for continuous variables) and chi-square or Fisher’s tests (categorical variables) were performed with Group (SA *vs* AC *vs* HC) as factor to test differences in sociodemographic and clinical variables. For variables showing significant differences in the three groups, simple contrast *post-hoc* tests were performed with False Discovery Rate (FDR) correction. Variables found to be significant with the *post-hoc* test were used as covariates in the analysis of the behavioral responses.

Then, two-way ANCOVAs for Group (SA *vs* AC *vs* HC) and Condition (Neutral *vs* Concordant *vs* Discordant) were performed using IG ambiguous and IG risky as dependent variables. The mean reaction time during the choices was calculated for each IGT phase, and the same analysis was done with reaction times as dependent variables. Simple contrast *post-hoc* tests were performed with FDR correction.

The alpha significance level was fixed at 0.05, and the threshold for significant trends at 0.07. All statistical analyses were performed with SPSS 20.0.

## Results

### Demographic and clinical variables

Sociodemographic variables, NART score and IGT explicit understanding were not significantly different in the three groups (all *p* > .05) (Table 1). As expected, the STAI state and trait scores were lower in the HC group than in the two patient groups. Moreover, HCs tended to report less frequently childhood emotional abuse and neglect compared with the two patient groups.

**Table 1:** Comparison of sociodemographic and clinical variables in healthy controls, affective controls and suicide attempters.

	Healthy controls (*n* = 30)	Affective controls (*n* = 41)	Suicide attempters (*n* = 45)	*p* values and post-hoc
*Sociodemographic data*				
Age	37.45 (1.54)	36.16 (1.29)	37.56 (1.29)	*p* = 0.698
Years of education	14.63 (.37)	14.49 (.31)	13.85 (.31)	*p* = 0.208
*Clinical characteristics*				
Age at first thymic episode	−	24.41 (1.27)	23.00 (1.29)	*p* = 0.436
Number of depressive episodes	−	3.24 (1.24)	6.28 (1.26)	*p* = 0.090
Number of manic episodes	−	1.83 (1.22)	2.72 (1.40)	*p* = 0.633
BDI score	0.50 (0.19)	4.44 (0.75)	4.73 (0.62)	*p* = 0.001, AC, SA > HC
HAMD score	1.33 (0.30)	3.93 (0.36)	3.84 (0.39)	*p* = 0.001, AC, SA > HC
YMRS score	0.03 (0.03)	0.44 (0.21)	0.53 (0.29)	*p* = 0.342
STAI state score	27.23 (1.05)	36.34 (1.64)	35.64 (1.53)	*p* = 0.001, AC, SA > HC
STAI trait score	31.13 (1.26)	44.10 (1.49)	46.69 (1.58)	*p* = 0.001, AC, SA > HC
BIS-10 total score	42.90 (1.96)	47.02 (2.12)	50.13 (2.11)	*p* = 0.069
IGT comprehension	0.92 (0.16)	0.63 (0.13)	0.64 (0.12)	*p* = 0.264
NART score	21.40 (0.75)	21.90 (0.64)	22.22 (0.61)	*p* = 0.696
*Lifetime psychiatric comorbidities*				
Bipolar disorder, *n* (%)	−	12 (29.3)	25 (55.6)	*p* = 0.012
Eating disorder, *n* (%)	−	4 (9.8)	7 (15.6)	*p* = 0.421
Anxiety disorder, *n* (%)	−	20 (48.8)	27 (60.0)	*p* = 0.297
PTSD, *n* (%)	−	2 (4.9)	5 (11.1)	*p* = 0.437
Alcohol/Substances abuse, *n* (%)	−	5 (12.2)	10 (22.2)	*p* = 0.221
*Suicidal history*				
Age at first suicide attempt	−	−	24.51 (3.28)	−
Number of suicide attempts	−	−	1.29 (.39)	−
Violent suicide attempt, Yes *n* (%)	−	−	2 (4.4)	−
Severe suicide attempt, Yes *n* (%)	−	−	9 (20.0)	−
*Medication*				
Benzodiazepines, Yes *n* (%)	−	1 (2.4)	7 (15.6)	*p* = 0.060
Antidepressants, Yes *n* (%)	−	9 (22.0)	7 (15.6)	*p* = 0.447
Antiepileptics, Yes *n* (%)	−	2 (4.9)	8 (17.8)	*p* = 0.093
Antipsychotics, Yes *n* (%)	−	1 (2.4)	7 (15.6)	*p* = 0.060
Lithium, Yes *n* (%)	−	3 (7.3)	4 (8.9)	*p* = 0.790
*Childhood trauma moderate/severe*				
CTQ physical abuse, Yes *n* (%)	1 (3.3)	3 (7.3)	3 (6.7)	*p* = 0.765
CTQ physical neglect, Yes *n* (%)	2 (6.7)	7 (17.1)	9 (20.0)	*p* = 0.278
CTQ emotional abuse, Yes *n* (%)	3 (10.0)	14 (34.1)	19 (42.2)	*p* = 0.011 AC, SA > HC
CTQ emotional neglect, Yes *n* (%)	4 (13.3)	11 (26.8)	22 (48.9)	*p* = .004 SA > HC
CTQ sexual abuse, Yes *n* (%)	1 (3.3)	6 (14.6)	9 (20.0)	*p* = 0.120

Data are shown as means ± SEM and frequencies.

*MDD* major depressive disorder, *BDI* beck depression inventory, *HAMD* Hamilton Depression Rating Scale, *YMRS* Young Mania Rating Scale, *STAI* State-Trait Anxiety Inventory, *BIS-10* Barrat Impulsiveness Scale, *IGT* Iowa Gambling Task, *NART* National Adult Reading Task, *PTSD* Post-traumatic stress disorder, *CTQ* Childhood Trauma Questionnaire, *HC* healthy controls, *AC* affective controls, *SA* suicide attempters.

In the two patient groups, bipolar disorder was more frequent in the SA than AC group (*p* < 0.012), while the other clinical variables were comparable (all *p* > 0.05).

Therefore, lifetime bipolar disorder was used as covariate in the behavioral analyses.

### Behavioral results

#### Reaction times for choices

**Fig. 2:**
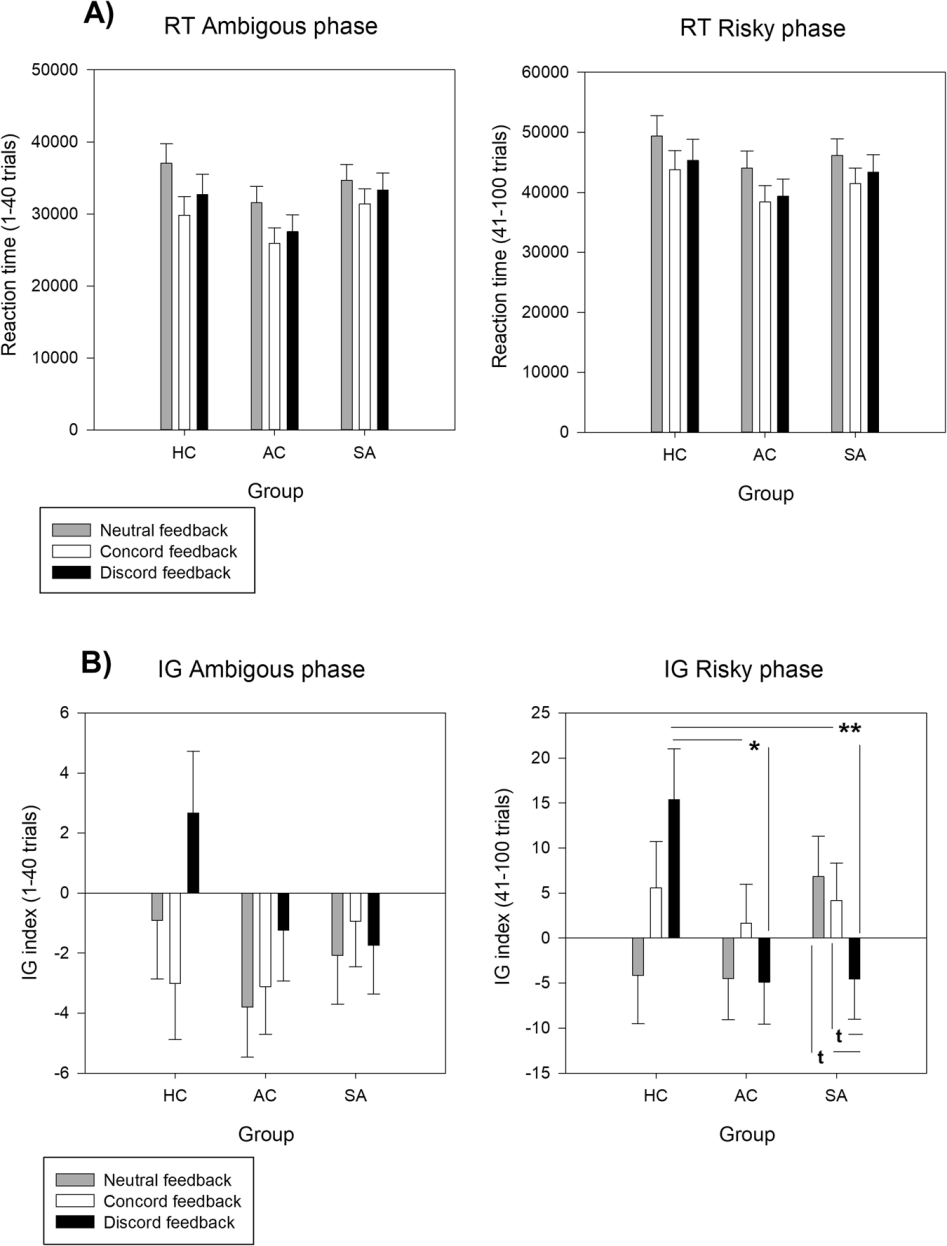
Behavioral results of ambigous and risky phases in the Iowa Gambling Task. **a** Total reaction time (RT) in the three groups and in the three emotional feedback conditions during the ambiguous (i.e., decision making under ambiguous uncertainty) and risky (i.e., decision making under risk uncertainty) phases of the Iowa Gambling Task. Data are the mean ± SEM of the total reaction time in seconds for the ambiguous and risky phases. **b** Performance (IG index) in the three groups and in the three emotional feedback conditions during the ambiguous (i.e., decision making under ambiguous uncertainty) and risky (i.e., decision making under risk uncertainty) phases of the Iowa Gambling Task. Group (SA *vs* AC *vs* HC) by condition (Neutral *vs* Concordant *vs* Discordant) interaction. Data are the mean ± SEM of the IG score for the ambiguous and risky phases. Note: **=*p* < 0.01, *=*p* < 0.05, ^t^=*p* < .07; HC healthy controls, AC affective controls, SA suicide attempters.

In the IGT ambiguous phase, there were significant Group differences (*F*_*2, 106*_ = 4.41*, p* < 0.014*, η*^*2*^ = .08*, 1 – β* = 0.76). Reaction times were faster in the AC group than in the SA (*p* < 0.029) and HC groups (*p* < 0.029*;* mean ± SEM: SA = 831.25 ± 40.99 ms, AC = 708.38 ± 32.43 ms, HC = 824.54 ± 32.99 ms). Moreover, reaction times were longer in participants who received neutral feedback than in participants who received concordant emotional feedback (mean ± SEM: Neutral = 860.83 ± 34.56 ms, Concordant = 725.95 ± 32.68 ms, Discordant = 777.38 ± 34.97 ms) (Condition factor: *F*_2, 106_ = 3.86, *p* < 0.024, *η*^*2*^ = 0.07, *power* = 0.70) (Fig. 2a).

In the IGT risky phase, there was a significant trend for the Condition factor (*F*_2, 106_ = 2.80, *p* < 0.065, *η*^*2*^ = 0.05, *power* = 0.55). However, *post-hoc* comparisons did not show any significant difference (mean ± SEM: Concordant = 686.36 ± 26.76 ms., Discordant = 707.96 ± 28.91 ms., Neutral = 775.78 ± 28.57 ms.) (Fig. 2a).

#### IGT performances

In the IGT ambiguous phase, no Group or Condition effect and no Group × Feedback interaction was observed (*p values* > 0.05) (Fig. 2b).

In the IGT risky phase, there was a significant Group × Condition interaction (*F*_*4, 106*_ = 2.75*, p* < 0.031*, η*^*2*^ = 0.09*, 1 – β* = 0.76). *Post-hoc* comparisons showed that the IG from the risky blocks was higher in the HC than AC (*p* < 0.023) and SA (*p* < 0.007) groups in the discordant feedback condition. Moreover, within the SA group, the IG from the risky blocks tended to be lower in patients in the discordant feedback condition than in the other two conditions (*p* < 0.061 for both) (Fig. 2b).

### Functional MRI

Table 2 summarizes the fMRI results during the IGT ambiguous and risky phases. Supplementary Fig. 2 shows the maps of brain activation without ROI masking during the ambiguous and risky phases. Supplementary Fig. 3 shows the comparison of ROI beta values in the three groups (HC, AC, and SA) during the risky vs. safe choice contrast.

**Table 2:** Significant group x emotion comparisons, voxel *p* < 0.001, k ≥ 10.

IGT ambiguous phase	K_E_	T	(x, y, z)	Selected ROI
Neutral > Concordant				
Suicide attempters	47	4.11	(− 50, 34, 10)	VLPFC (L)
	26	4.04	(56, 12, 4)	VLPFC (R)
Affective controls	26	4.09	(6, 32, 24)	ACC (R)
	25	4.11	(− 4, 20, 32)	ACC (L)
	21	4.16	(36, 18, − 6)	OFC (R)
	19	4.03	(− 22, 30, 48)	DPFC (L)
	15	3.57	(50, 26, 32)	DPFC (R)
	14	3.75	(6, 60, − 2)	OFC (R)
	11	4.29	(− 2, 8, 50)	ACC (L)
	10	3.70	(− 12, 60, − 4)	OFC (L)
Healthy controls	69	4.00	(42, 50, − 4)	OFC (R)
	18	3.71	(0, 28, 24)	ACC (L)
Neutral > Discordant				
Healthy controls	99	4.04	(32, 46, 20)	DPFC (R)
	16	3.48	(− 50, 34, 6)	VLPFC (L)
	13	3.90	(42, 48, 0)	DPFC (R)
IGT risky phase	K_E_	T	(x, y, z)	ROI selected
Discordant > Neutral				
Suicide attempters	39	4.31	(36, 24, 52)	DPFC (R)
	10	3.78	(− 26, 16, − 16)	OFC (L)
Neutral > Concordant				
Affective controls	41	4.12	(4, 30, − 4)	ACC (R)
	27	3.84	(− 34, 44, 20)	DPFC (L)
	14	3.61	(− 24, 56, 2)	MPFC (L)

*IGT* Iowa Gambling Task, *ROI* Regions of interest; *(L)* Left, *(R)* Right, *VLPFC* Ventrolateral prefrontal cortex, *ACC* Anterior cingulate cortex, *OFC* Orbitofrontal cortex, *DPFC* Dorsal prefrontal cortex, *MPFC* Medial prefrontal cortex.

#### Risky vs. Safe choices during the ambiguous IGT phase

**Fig. 3:**
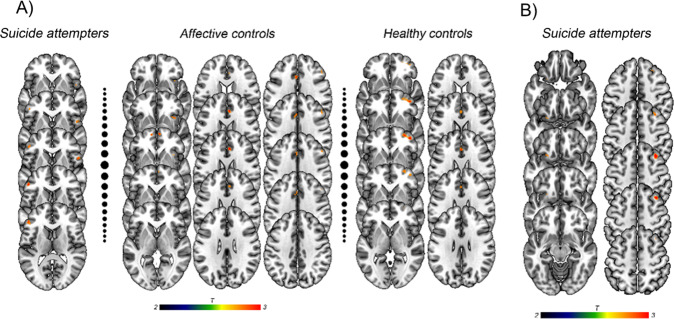
Brain activation during risky (A and B decks) vs. safe (C and D decks) choices in the Iowa Gambling Task. **a** Neutral > Concordant emotional feedback condition for suicide attempters, affective controls and healthy controls during the ambiguous phase. **b** Discordant > neutral emotional feedback condition for suicide attempters during the risky phase.

In the concordant vs. neutral feedback condition contrast, bilateral VLPFC activation was reduced in the SA group (Left: *p* < 0.001, *k* = 47; Right: *p* < 0.001, *k* = 26); bilateral activation of ACC (Left: *p* < 0.001, *k* = 25; Right: *p* < 0.001, *k* = 26), OFC (Left: *p* < 0.001, *k* = 10; Right: *p* < 0.001, *k* = 21) and DPFC (Left: *p* < 0.001, *k* = 19; Right: *p* < 0.001, *k* = 15) was decreased in the AC group; and activation of the left ACC (*p* < 0.001, *k* = 18) and right OFC (*p* < 0.001, *k* = 69) was reduced in the HC group (Fig. 3a).

In the discordant vs. neutral feedback condition contrast, activation was decreased in the right DPFC (*p* < 0.001, *k* = 99) and left VLPFC (*p* < 0.001, *k* = 16) in the HC group.

#### Risky vs. Safe choices during the risky IGT phase

In the concordant vs. neutral feedback condition contrast, activation of the right ACC (*p* < 0.001, *k* = 41) and left DPFC (*p* < 0.001, *k* = 27) and MPFC (*p* < 0.001, *k* = 14) was reduced in the AC group.

In the discordant vs. neutral feedback condition contrast, activation was increased in the right DPFC (*p* < 0.001, *k* = 39) and left OFC (*p* < 0.001, *k* = 10) in patients with SA (Fig. 3b).

#### Correlations between fMRI and behavioral data

The ROI beta values of each patient for the ambiguous and risky phases were extracted. Pearson’s correlations between the beta values and the behavioral data (reaction time and IG index) showed significant negative correlations between the IG index (CD – AB) during the risky phase and the activation of all the brain areas (all *p* < 0.05 FDR corrected). Thus, higher activation in prefrontal areas was associated with lower preference for the safe decks (lower IG index) during the risky phase (see Table 1 in supplementary material).

## Discussion

The present study showed that during a decision-making task that included emotional feedback, behavioral performance and brain activation patterns are different in euthymic suicide attempters compared with controls (AC and HC).

We did not found significant effects of condition (congruent, incongruent or neutral feedback) at behavioral level. It is not on line with results from Aite et al.^25^ where, conducted, in healthy controls. Congruent feedback was associated with safety choices whereas incongruent feedback was associated with risky choices during IGT. This contradiction may be explained by several reasons. First, our sample included patients with history of suicide attempt, characterized by difficulties in decision making^20^. Second, the lack of power in healthy subjects may explain the lack of results for this sub-group. Third, while Aite et al.^25^ used fearful faces for the negative feedback, we used angry faces.

During the IGT ambiguous phase, the IG index was not different in the three groups, whereas concordant feedback was associated with slower reaction times in the SA group, as previously reported by Chen et al.^24^. They showed that congruence between emotional and monetary outcomes lowered the reaction times during decision making^24^. Moreover, another study found that healthy women tend to take more risks after concordant feedback (monetary losses combined with angry faces)^39^.

Our neuroimaging results may suggest that suicide attempters did not correctly benefit from congruent emotional feedback during the IGT phase of ambiguous uncertainty. In both control groups, activation of OFC and ACC was reduced during risky vs. safe choices in the concordant vs. neutral feedback condition. Regulation of emotional conflicts (i.e., processing of task-relevant stimuli in conflict with incongruent emotional information) increases ACC activity^40^. A decrease in ACC suggests that congruent feedback facilitates emotional conflict resolution. In parallel, OFC implication decreased because congruent feedback might also have facilitated the attribution of value and risk during decision making^17^. Conversely, activity in these areas was not decreased in suicide attempters. This might suggest that differently from controls, suicide attempters did not process concordant feedback. On the other hand, VLPFC activation when receiving concordant feedback during the risky vs. safe choices was reduced only in suicide attempters, in agreement with a previous study showing a decrease in VLPFC activation in suicide attempters during risky vs. safe choices^14^.

During the IGT risky phase, the performance of suicide attempters in the discordant feedback condition was worse than that of healthy controls and also compared with their own results in the neutral and concordant feedback conditions. This result might suggest altered performances under risky uncertainty with discordant feedback in suicide attempters. Discordant feedback may increase the probability of choosing risky decks in order to avoid angry faces that have higher salience in suicide attempters than healthy controls. At the neuroanatomical level, activation of OFC and DPFC was higher in suicide attempters for risky vs. safe choices in the discordant feedback compared with the neutral feedback condition. The incongruence between emotional feedback and choice (independently of the monetary feedback) may increase the executive control required to understand the decision-making task and to pick cards from the advantageous decks. Moreover, previous research using emotional images to create interference during a Stroop task showed that emotional negative information disturbed the executive control and increased OFC activation^41^. Suicide attempters are characterized by deficits in executive functions linked to the prefrontal areas, such as inhibitory control and working memory^42,43^. The increase in DPFC activation observed in suicide attempters but not in controls may reflect an impaired emotional-cognitive regulation due to incongruent emotional information^20^.

Our study has some limitations. First, the small size of the different groups increased the risk of type-I and type-II errors. This limits the application of stringent corrections^44^ and the adjustment for several variables. However, this was the first study on the interaction between emotional and monetary feedback in a clinical sample of suicide attempters. Second, ongoing pharmacological treatments also might have influenced brain activation in patients. However, medication load was comparable between patients’ groups. Third, only women were included in the study. This did not allow assessing the gender effect and limits the generalization of our findings.

Nonetheless, our study proposes a new approach to investigate how socially relevant information might influence decision-making in suicide attempters. These results strengthen the evidence for a specific deficit in prefrontal cortex activity during decision making that may be part of the suicidal diathesis. Our behavioral results suggest that patients with suicidal vulnerability avoid negative social feedback, despite the expected outcome. It has been demonstrated that dysfunctional evaluation of the social context is an important marker of suicidal vulnerability^27^. Our study shows that in suicide attempters, both prefrontal brain activity and behavior are altered during decision making in the presence of social feedback. It suggests that emotional feedback does not have the same effect in suicide attempters and in affective and healthy controls. Consequently, suicide attempters may be more vulnerable to social stress^45^, increasing the risk of more deleterious decisions. Thus, patients with history of suicidal acts might benefit from therapies that focus on emotion visualization and interpretation to better adapt to the challenges of daily life.

## Supplementary information

Supplemental material
