# Supplementary material for: Prefrontal activation in suicide attempters during decision making with emotional feedback
Source: Transl Psychiatry. 2020 Sep 18;10:313. doi: 10.1038/s41398-020-00995-z (PMC7501865; doi:10.1038/s41398-020-00995-z)
Supplement: Supplementary file 1 — Supplemental material [file 41398_2020_995_MOESM1_ESM.docx]

**Supplementary material Table 1: Pearson correlations between the beta values of the Regions-of-interest and behavioral data in the whole sample for the ambiguous and the risky phases:**

|  | Ambiguous | | Risky | |
| --- | --- | --- | --- | --- |
|  | IG index | Reaction time | IG index | Reaction time |
| Left ACC | r = .136 | r = .048 | **r = - .355^**^** | r = .140 |
| Right ACC | r = .087 | r = .079 | **r = - .297^**^** | r = .102 |
| Left DPFC | r = .064 | r = .027 | **r = - .354^**^** | r = .178 |
| Right DPFC | r = .015 | r = .060 | **r = - .377^**^** | r = .136 |
| Left MPFC | r = - .022 | r = .049 | **r = - .291^**^** | r = .111 |
| Right MPFC | r = .033 | r = .074 | **r = - .345^**^** | r = .057 |
| Left OFC | r = .111 | r = .022 | **r = - .272^**^** | r = .033 |
| Right OFC | r = .089 | r = .029 | **r = - .271^**^** | r = - .038 |
| Left VLPFC | r = .034 | r = .017 | **r = - .206^*^** | r = .022 |
| Right VLPFC | r = .121 | r = .065 | **r = - .235^*^** | r = .018 |

Note: *p < .05; **p < .01; all p values after correction for multiple comparisons using the False Discovery Rate (FDR); IG = Iowa Gambling; ACC = Anterior cingulate cortex; DPFC = Dorsal prefrontal cortex; MPFC = Medial prefrontal cortex; OFC = Orbitofrontal cortex; VLPFC = Ventrolateral prefrontal cortex.


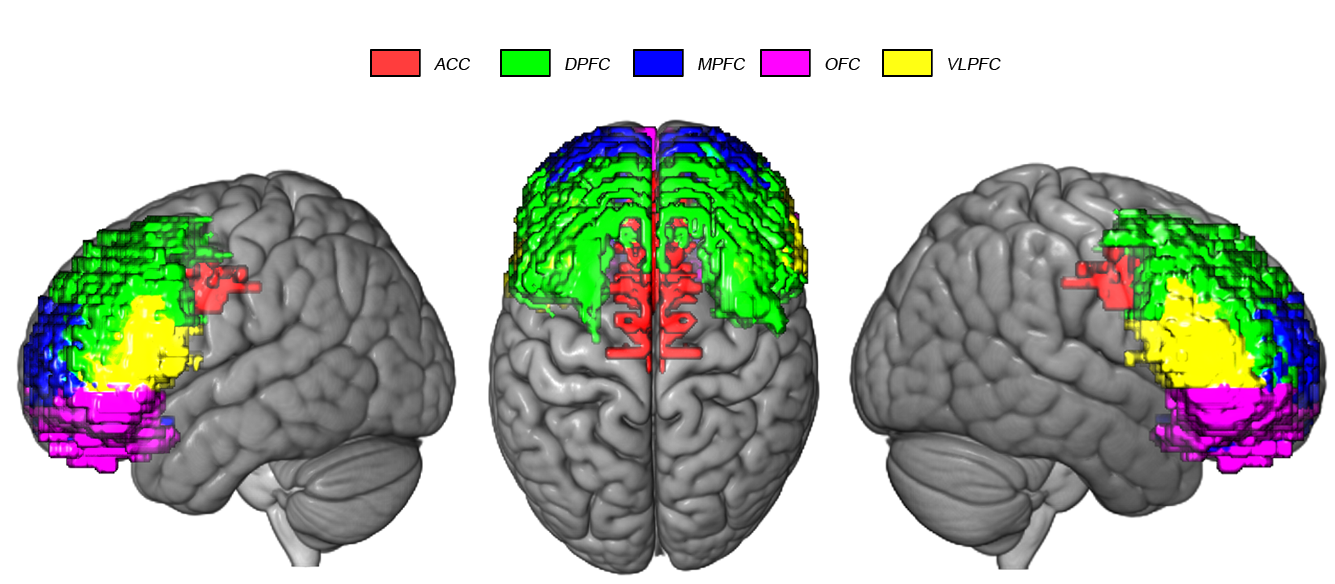
**Supplementary Figure 1: Representation of the five Regions-of-interest (ROI) under study:**

Note: ACC = Anterior cingulate cortex; DPFC = Dorsal prefrontal cortex; MPFC = Medial prefrontal cortex; OFC = Orbitofrontal cortex; VLPFC = Ventrolateral prefrontal cortex

**Supplementary Figure 2: Whole brain activation during risky (A and B decks) vs. safe (C and D decks) choices in the Iowa Gambling Task (whole sample). A) Activation during the ambiguous phase; B) Activation during the risky phase:**

1. **Ambiguous B) Risky**


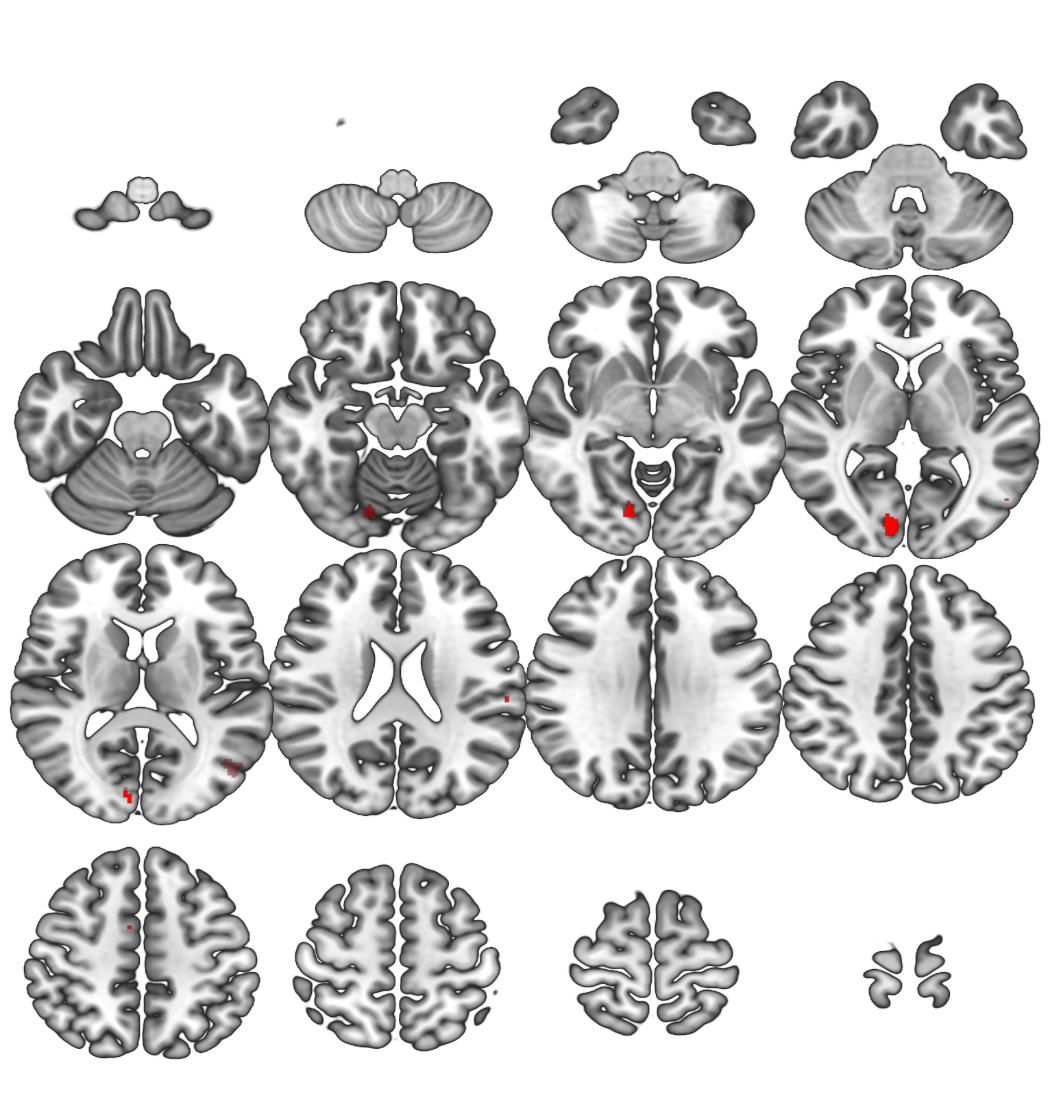

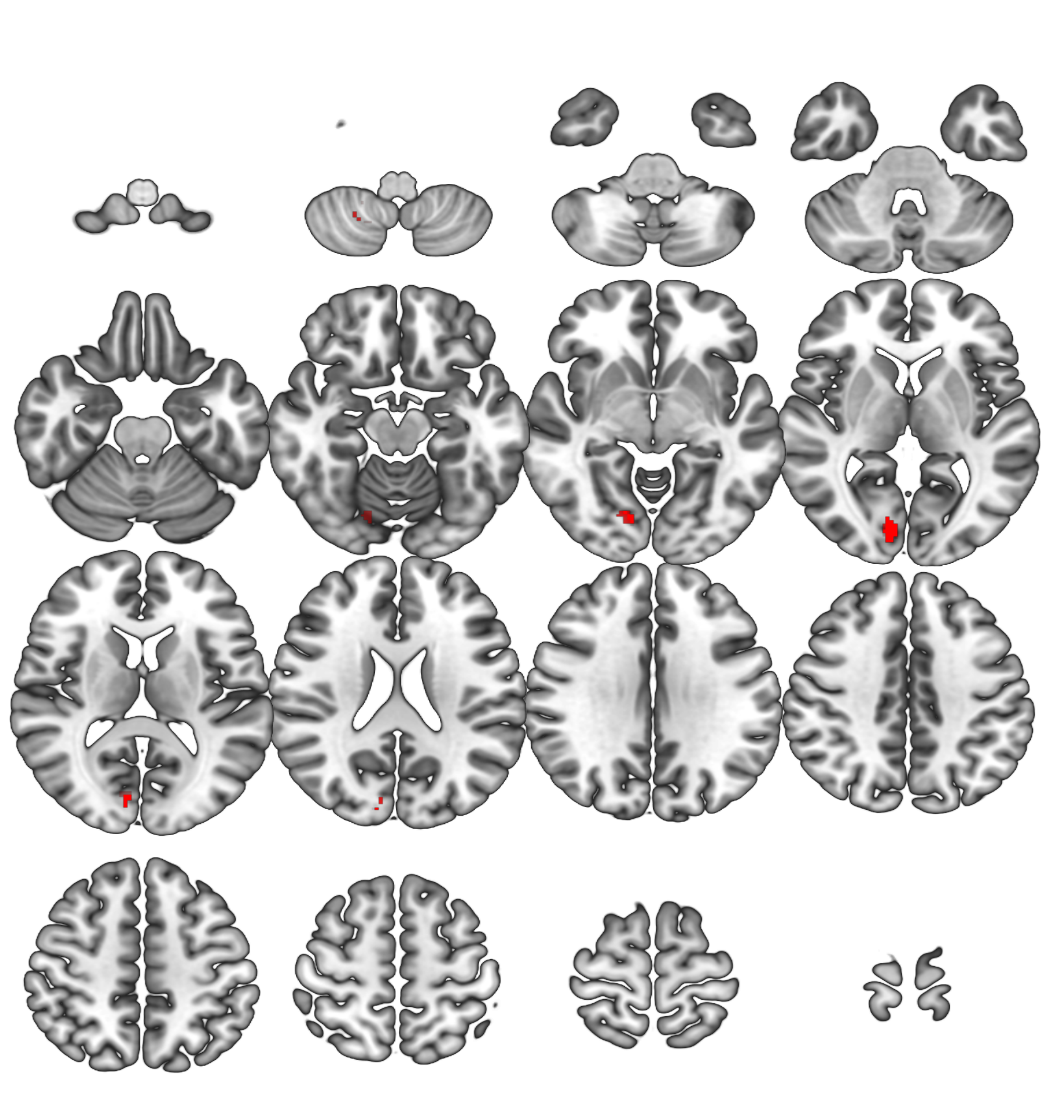


**Supplementary Figure 3: Beta values of the Regions-of-interest (ROI), shown as median ± 95 % confidence intervals (CI) for each group: A) For the contrast AB > CD in the ambiguous phase; B) For the contrast AB > CD in the risky phase:**

1. **Ambiguous**


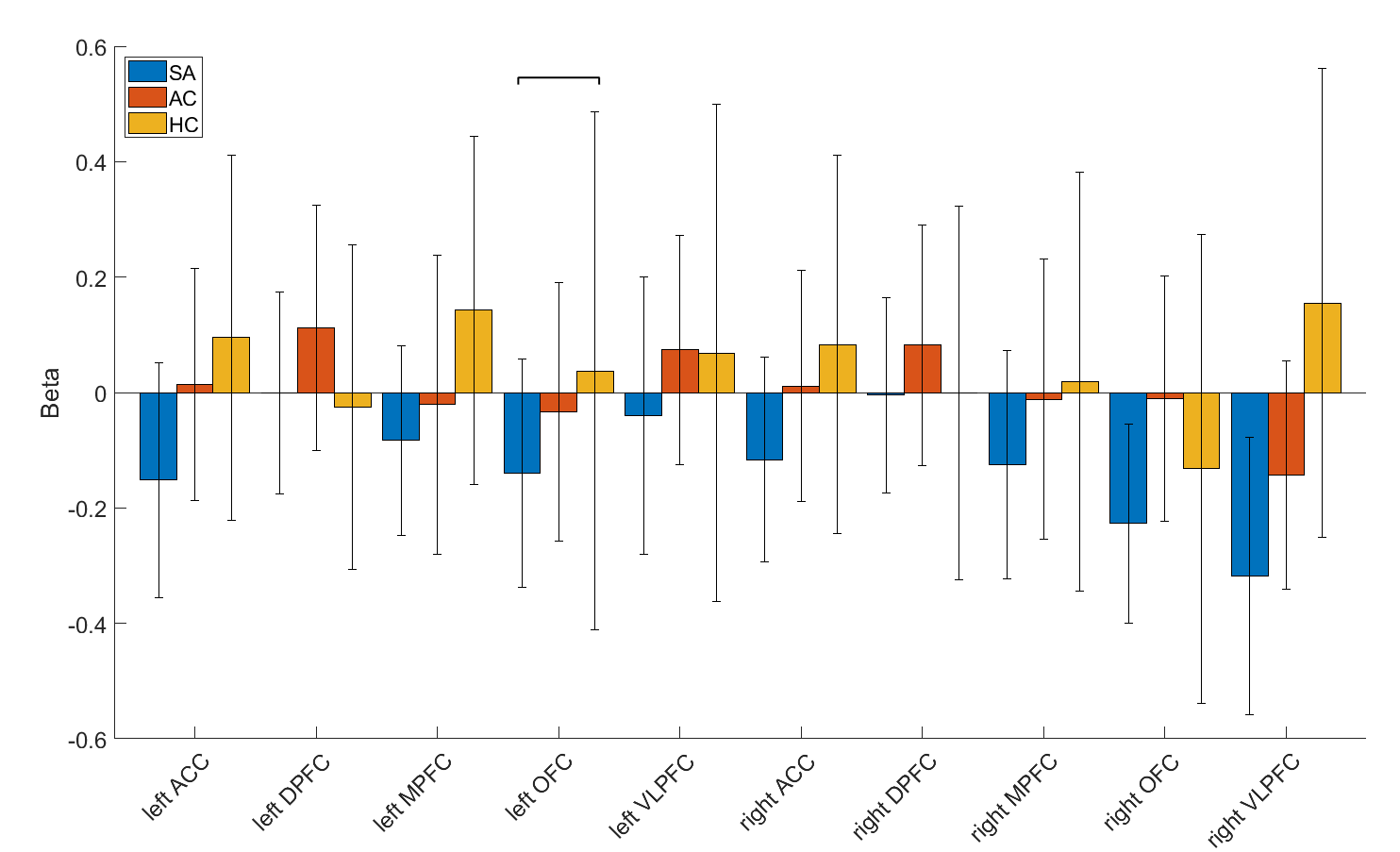


1. **
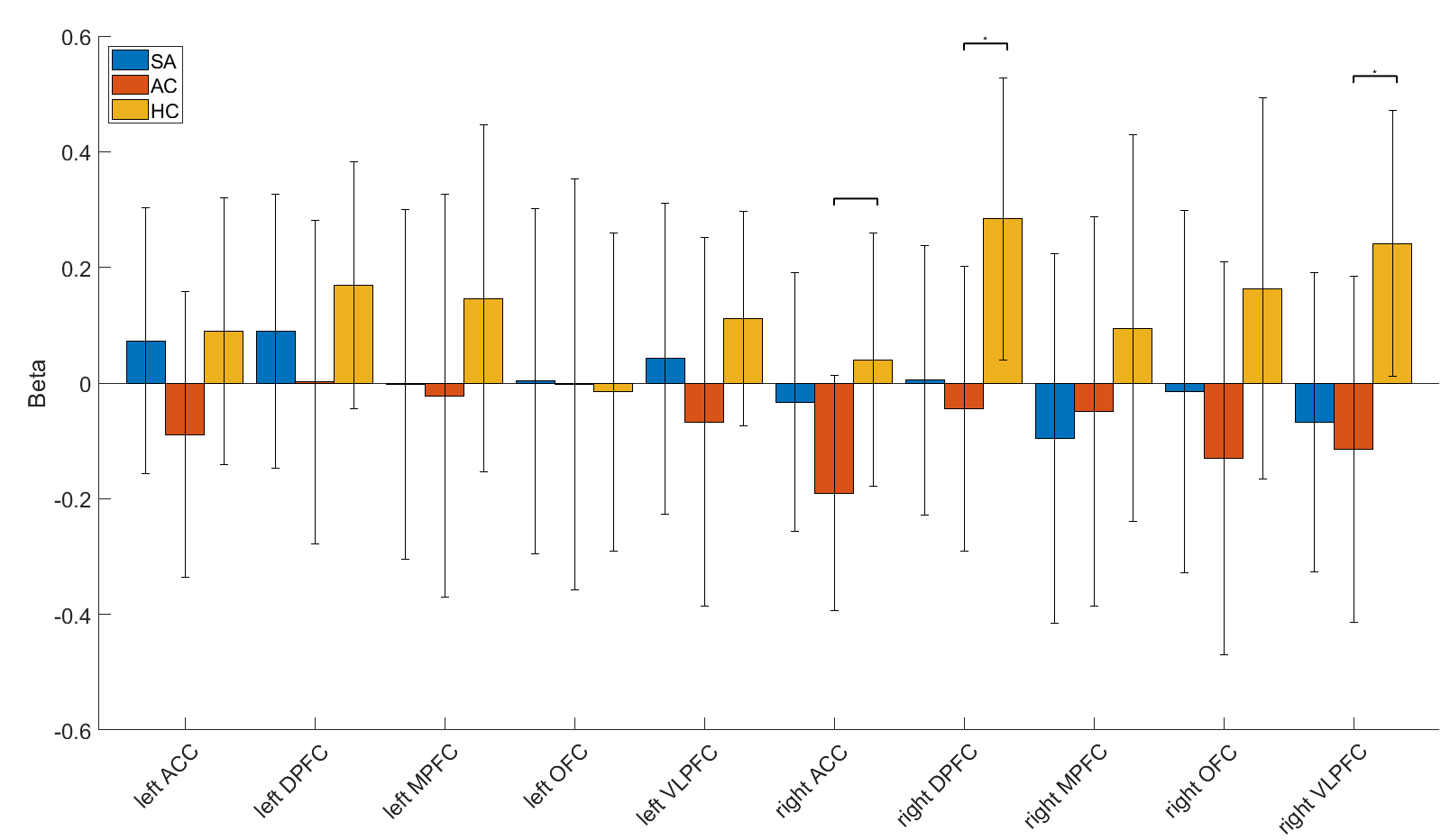
Risky**

Note: *p < .05; without *p < .10; after correction for multiple comparisons, none of the differences remained significant (p>0.05 FDR); ACC = Anterior cingulate cortex; DPFC = Dorsal prefrontal cortex; MPFC = Medial prefrontal cortex; OFC = Orbitofrontal cortex; VLPFC = Ventrolateral prefrontal cortex.
